# Optimized method for extraction of exosomes from human primary muscle cells

**DOI:** 10.1186/s13395-020-00238-1

**Published:** 2020-07-08

**Authors:** Laura Le Gall, Zamalou Gisele Ouandaogo, Ekene Anakor, Owen Connolly, Gillian Butler Browne, Jeanne Laine, William Duddy, Stephanie Duguez

**Affiliations:** 1 Northern Ireland Center for Stratified/Personalised Medicine, Biomedical Sciences Research Institute, Ulster University, Derry~Londonderry, UK; 2 Centre for Research in Myology, INSERM UMRS_974, Sorbonne Université, Paris, France

**Keywords:** Extracellular vesicle, Muscle exosome extraction in vitro, Muscle secretome

## Abstract

Skeletal muscle is increasingly considered an endocrine organ secreting myokines and extracellular vesicles (exosomes and microvesicles), which can affect physiological changes with an impact on different pathological conditions, including regenerative processes, aging, and myopathies. Primary human myoblasts are an essential tool to study the muscle vesicle secretome. Since their differentiation in conditioned media does not induce any signs of cell death or cell stress, artefactual effects from those processes are unlikely. However, adult human primary myoblasts senesce in long-term tissue culture, so a major technical challenge is posed by the need to avoid artefactual effects resulting from pre-senescent changes. Since these cells should be studied within a strictly controlled pre-senescent division count (<21 divisions), and yields of myoblasts per muscle biopsy are low, it is difficult or impossible to amplify sufficiently large cell numbers (some 250 × 10^6^ myoblasts) to obtain sufficient conditioned medium for the standard ultracentrifugation approach to exosome isolation.

Thus, an optimized strategy to extract and study secretory muscle vesicles is needed. In this study, conditions are optimized for the in vitro cultivation of human myoblasts, and the quality and yield of exosomes extracted using an ultracentrifugation protocol are compared with a modified polymer-based precipitation strategy combined with extra washing steps. Both vesicle extraction methods successfully enriched exosomes, as vesicles were positive for CD63, CD82, CD81, floated at identical density (1.15-1.27 g.ml^−1^), and exhibited similar size and cup-shape using electron microscopy and NanoSight tracking. However, the modified polymer-based precipitation was a more efficient strategy to extract exosomes, allowing their extraction in sufficient quantities to explore their content or to isolate a specific subpopulation, while requiring >30 times fewer differentiated myoblasts than what is required for the ultracentrifugation method. In addition, exosomes could still be integrated into recipient cells such as human myotubes or iPSC-derived motor neurons.

Modified polymer-based precipitation combined with extra washing steps optimizes exosome yield from a lower number of differentiated myoblasts and less conditioned medium, avoiding senescence and allowing the execution of multiple experiments without exhausting the proliferative capacity of the myoblasts.

## Introduction

In addition to its classical role in locomotion, skeletal muscle is increasingly recognized to have a role in signaling via its secretory functions. Interleukin-6 (IL-6) [1] and musculin [2] have been identified to originate and be secreted from skeletal muscle in vivo, and the secretomic profiles of muscle cells in vitro, such as C_2_C_12_ myotubes [3, 4], human myotubes [5], and rat muscle explants [6] include growth factors (e.g., follistatin-like protein 1, IGF2, TGF), cytokines, and inhibitors of collagenase (e.g., TIMP2). These studies suggest that skeletal muscle can be viewed as an endocrine organ. Secreted proteins—also named myokines [2]—may act in an autocrine/paracrine manner on muscle cells or other types of cell and contribute to muscle growth and regeneration, body-wide metabolism, and other functions [see [7] for review].

In addition to proteins exiting the cell by classical secretory pathways, muscle cells also release protein-associated vesicles [5]. These extracellular vesicles (EVs) are widely studied in different physiological and pathological contexts, and are known to play a key role in tissue homeostasis [8], embryogenesis and development [9], cell survival [10], inflammatory and metabolic diseases [11, 12], cancer metastasis [13]. EVs are broadly classified as exosomes, ectosomes, or apoptotic bodies. Exosomes (40–120 nm) are formed from the endolysosomal pathway and are released into the extracellular space when multivesicular bodies containing intraluminal vesicles undergo exocytosis [14]. Ectosomes (100–1000 nm) encompass microvesicles, microparticles, or shedding vesicles and are formed from the direct budding of the plasma membrane [15]. Finally, apoptotic bodies (500–2000 nm) result from the outward bulge of the cell membrane due to cytoskeleton dysfunction and usually contain a part of the cytoplasm [16]. Human skeletal muscle cells are known to secrete two categories of veisicle, exosomes, and microvesicles [5]. Both types of muscle cell vesicles can fuse and deliver functional proteins into target cells, as shown by the delivery of alkaline phosphatase through vesicles to human dermofibroblasts that do not have an endogenous activity for alkaline phosphatase [5]. Exosomes and microvesicles from other cell types have been described to play a role in intercellular communication and to induce physiological changes in recipient cells, such as induction of cellular oncogenic transformation [17] or T-cell activation [18]. While the role of cytokines (e.g., [19–21]) and vesicles (e.g., [18, 22]) originating from inflammatory cells is well documented, the role of their secretion by myoblasts or differentiating myotubes is relatively unexplored, particularly concerning regenerative processes in injury and aging, and inflammatory and fibrotic processes in various muscle pathologies. Primary human myoblasts obtained from muscle biopsies are an invaluable in vitro tool for studying a pure human muscle secretome but this poses a technical challenge relating to the volume of conditioned media required per data point and their limited proliferative capacity [23]. Since primary human myoblasts should be studied within a strictly controlled pre-senescent division count (<21 divisions), and yields of myoblasts per muscle biopsy can be low, it can be difficult or impossible to amplify sufficiently large cell numbers (some 250 × 10^6^ myoblasts) to obtain sufficient conditioned medium for certain approaches to exosome isolation.

The isolation of exosomes from cell culture have been achieved by ultracentrifugation-based methods [24, 25], size-based techniques [24, 26, 27], polymer-based precipitation [28], and immunoaffinity capture-based techniques [24]. Ultracentrifugation is considered the gold standard and is the most reported exosome isolation technique [29]. However, ultracentrifugation has several shortcomings including the need for a large volume of biological fluid or conditioned cell culture media, long run-time, and limited reproducibility [30].

In this study, we highlight the challenges surrounding the study of vesicles secreted by primary human muscle cells and we compare two strategies—(1) ultracentrifugation-based isolation and (2) a modified polymer-based precipitation approach—in terms of quality and yield of exosomes. We define an optimized protocol to extract exosomes from primary muscle cells, without exhausting the number of pre-senescent divisions and thereby enabling a larger number of experiments to be carried out on a given cell line.

## Materials and methods

### Primary cell extractions

Six deltoid muscle biopsies were obtained from ALS patients (50.0 ± 6.5 years old) who attended the Motor Neuron Diseases Center (Pitié Salpétrière, Paris), and 17 muscle biopsies from healthy subjects (51.4 ± 18.2 years old) from the BTR (Bank of Tissues for Research, a partner in the EU network EuroBioBank) in accordance with European recommendations and French legislation. The protocol (NCT01984957) was approved by the local Ethical Committee. Written informed consent was obtained from all patients. All biopsies were isolated from the deltoid muscle.

### Cell culture proliferation and differentiation

Primary human myoblasts were extracted from fresh muscle biopsies as described previously [31]. Briefly, myoblasts were sorted using CD56 magnetic beads (Milteny®) and expanded in 0.22-μm filtered proliferating medium containing DMEM/M199 medium supplemented with 20% FBS, 25 μg/ml Fetuin, 0.5 ng/ml bFGF, 5 ng/ml EGF, 5 μg/ml insulin and incubated at 5%CO_2_, 37 °C. The number of cell divisions was calculated using the formula below. The myogenicity of the culture was determined by counting the number of nuclei positive for desmin against the total number of nuclei using the primary antibody anti-desmin (D33, 1:100, Dako). The secondary antibody was goat anti-mouse IgG1 AlexaFluor 594 (1:400, Invitrogen™), and counterstaining was performed with 1 μg.ml^−1^ DAPI as described below. After CD56 MACS sorting, 91.78 ± 8.32% of the cells were myogenic.

$$ \mathrm{Division}\ \mathrm{number}=\frac{\log \left(\frac{\mathrm{Cell}\ \mathrm{number}\ \mathrm{at}\ \mathrm{day}\ n}{\mathrm{Cell}\ \mathrm{number}\ \mathrm{plated}}\right)}{\log 2} $$

For differentiation into myotubes, 7.5 × 10^6^ myoblasts were plated in 225 cm^2^ flask (Falcom™) and let adhere overnight. Seeded myoblasts were then washed six times with supplement free DMEM and differentiated in DMEM for 72 h. The conditioned medium was then collected and used for exosome extraction.

### Beta-galactosidase staining

The senescence level was assessed using a Senescence β-Galactosidase Staining Kit (Cell Signaling Technology®).

### Cell immunostaining

The cells were fixed with 3.6% formaldehyde, permeabilized, blocked, and stained as described previously [32]. Primary antibody anti-myosin heavy chain (MF20, 1:50, DSHB) and secondary antibody goat anti-mouse IgG2b AlexaFluor 594 (1:400, Invitrogen™) were used to determine the formation of myotubes. The slides were washed and counter-stained with 1 μg.ml^−1^ DAPI for 2 min and then rinsed twice with PBS before being mounted with ibidi mounting medium (ibidi®).

### Protein extraction from cells

Myoblasts were scraped into 50 μl of chilled RIPA lysis buffer (Invitrogen™) supplemented with 1× Halt™ protease inhibitor cocktail (Thermo Scientific™) and incubated on ice for 10 min. Cell lysates were then centrifuged at 14,000*g* for 10 min at 4 °C and protein supernatants were collected and stored at −80 °C for downstream SDS-PAGE and immunoblotting.

### Condition culture media clearance

At the time of collection, the conditioned medium is centrifuged at 200*g* for 10 min. The subsequent supernatant was then centrifuged at 4000*g* for 20 min. The resulting supernatant was centrifuged for 70 min at 4 °C at 20,000*g* and then filtered through a 0.22-μm filter. The cleared medium was then stored at −80 °C prior to exosome extraction.

### Muscle exosome extraction using ultracentrifugation

Cleared media were centrifuged at 100,000*g* for 70 min at 4 °C following a method described previously [24]. The subsequent pellet was resuspended in PBS and washed three times by centrifugation at 100,000*g* for 70 min at 4 °C. The clean pellet was then resuspended in 100 μl of PBS or in NuPAGE™ LDS sample buffer for Western blot experiments.

### Exosome extraction using polymer precipitation

Cleared culture media was mixed with the Total Exosome Isolation kit (LifeTechnologies™) at a 2:1 volume ratio and incubated at 4 °C overnight. The mixture was then centrifuged at 10,000*g* for 60 min at 4 °C. The subsequent pellet was resuspended in 500 μl of PBS and washed three times using 100 kDa Amicon® filter column. The exosomes were then resuspended in 100 μl of PBS or in NuPAGE™ LDS sample buffer for Western blot experiments.

### Exosome protein extraction

Exosomes were lysed in 8 M urea supplemented with 1× Halt™ Protease Inhibitor cocktail (Thermo Scientifc™) and 2% SDS. Samples were incubated at 4 °C for 15 min, and exosome lysates were centrifuged at 14,000*g* for 10 min at 4 °C. Supernatants containing soluble proteins were stored at −80 °C.

### SDS-PAGE and Western blotting

SDS-PAGE was performed as follows. For cell lysates, protein concentrations were measured at 562 nm using the bicinchoninic acid assay kit (Pierce™) and 20 μg of protein was mixed with 4× NuPAGE™ LDS sample buffer. For exosome extracts, proteins were also mixed with 4× NuPAGE™ LDS sample buffer. For reducing conditions, samples were supplemented with 10× NuPAGE™ reducing agent. For the immunoblotting of tetraspanins, samples were prepared similarly but for the omission of reducing agents. All samples were then denatured at 70 °C for 10 min before being added to a 4–12 % polyacrylamide Bis-Tris gel (Life Technologies™) and electrophoresed at 200 v for 70 min in MOPS SDS Running buffer (LifeTechnologies™). Following electrophoresis, the gel was incubated in 20% ethanol for 10 min and proteins were transferred onto polyvinylidene fluoride membrane using the iBlot™ 2 Dry Blotting system (LifeTechnologies™) according to manufacturer’s instructions.

Immunoblotting was performed using the iBind™ Flex western system following the manufacturer’s instructions (Life Technologies™). PVDF membrane was probed with primary antibodies forPARP-1 (9542, Cell Signaling, rabbit IgG, 1:1000), or CD63 TS63 (10628D, Life Technologies™, mouse, 2 μg/ml), or CD81 (MA5-13548, Life Technologies™, mouse IgG, 1:100, v:v dilution), Flotillin (PA5-18053, Life Technologies™, 0.3 μg/ml) or HSPA8 (MABE1120, Millipore, mouse IgG, 1:1000 ) or Alix (SC-53540, Santa Cruz, 1:1000) and Goat anti-mouse or Goat anti-rabbit secondaries conjugated with HRP (LifeTechnologies™, 1:400, and 1:10,000 respectively). The membrane was then incubated with Amersham ECL Prime Western Blotting Detection Reagent for 5 minutes at room temperature and images were subsequently acquired using the UVP ChemiDoc-It™2 Imager and UVP software.

### Electron microscopy and immunogold

Extracted and further whole-mounted vesicles were processed as described in [24]. Observations were made using a CM120 transmission electron microscope (Philips, Eindhoven, The Netherlands) at 80 kV and images recorded with a Morada digital camera (Olympus Soft Imaging Solutions GmbH, Münster, Germany).

### Determination of the exosome density

Exosomes extracted from the cell culture medium using either ultracentrifugation or polymer-based precipitation were resuspended in 100 μl of PBS and loaded on the top of the sucrose gradient as previously described [5, 32]. Samples were then centrifuged at 100,000*g* for 17 h at 4 °C. Twelve fractions were sequentially collected, diluted in 12 ml PBS and centrifuged at 100,000*g* for 70 min at 4 °C. Each pellet was then resuspended in non-reducing NuPAGE™ LDS sample buffer and used for western blot analyses as described above. The density gradient of each fraction was determined using the method described by [33] by measuring the absorbance at 244 nm:

$$ \mathrm{Density}\left(\mathrm{g}.{\mathrm{cm}}^{\hbox{-} 3}\right)=\frac{\mathrm{Absorbance}\;\mathrm{at}244\;\mathrm{nm}+5.7283}{5.7144} $$

### Nanoparticle tracking analysis (NTA)

Exosome pellets were resuspended in 100 μl of filtered PBS. The exosome suspension was then diluted 10× in PBS. The size and distribution of exosomes secreted by primary muscle cells were evaluated by a NanoSight LM10 instrument (NanoSight) equipped with NTA analytic software (version 2.3 build 2.3.5.0033.7-Beta7). Three videos of 30 s were as previously described [34, 35] at the temperature set to 22.5 °C. The minimum particle size, track length, and blur were set to “automatic”.

### Proteomic analysis

- The exosome pellets were re-suspended in 25 μl 8 M Urea, 50 mM ammonium bicarbonate, pH 8.5, and reduced with DTT for 1 h at 4 °C. Protein concentrations were then quantified using Pierce BCA Protein Assay kit (ThermoFisher®). Exosomal proteins were kept at −80 °C.
- Proteome profile determined by mass spectrometry—20 μg of exosome protein were trypsin digested using a SmartDigest column (Thermo) for 2 h at 70 °C and centrifugated at 1400 rpm. Peptides were then fractionated into 8 fractions using a high pH reverse phase spin column (Thermo). Fractioned peptides were vacuum dried, resuspended, and analysed by data-dependent mass spectrometry on a Q Exactive HF (Thermo) with the following parameters: Positive Polarity, m/z 400-2000 MS Resolution 70,000, AGC 3e6, 100 ms IT, MS/MS Resolution 17,500, AGC 5e5, 50 ms IT, Isolation width 3 m/z, and NCE 30, cycle count 15.
- Database search and quantification—The MS raw data sets were searched for protein identification for semi-tryptic peptides against the Uniprot human database for semi tryptic peptides including common contaminants, using MaxQuant software (version 1.6.2.1) (https://wSww.biochem.mpg.de/5111795/maxquant). We used default parameters for the searches: mass tolerances were set at ±20 ppm for first peptide search and ±4.5 ppm for main peptide search, maximum two missed cleavage, and the peptide and resulting protein assignments were filtered based on a 1% protein false discovery rate (thus 99% confidence level). A total of 1254 proteins were detected in at least 1 sample. The mass spectrometry proteomics data have been deposited to the ProteomeXchange Consortium via the PRIDE partner repository with the dataset identifier PXD015736.
- To test for overlap with known exosome proteins from previous studies, all proteins detected in at least 1 proteomic sample were entered into the Funrich tool for vesicle functional analysis [36–39], and a Venn diagram generated against the subset of the Vesiclepedia database comprising previously observed exosomal proteins detected by mass spectrometry in human samples.

### mRNA extraction from polymer precipitated exosomes

Exosomes were first dissolved in 900 μl TRIzol® (Invitrogen™), then 200 μl of chloroform was added. After 5 min of incubation at RT, samples were centrifuged at 12,000*g* for 15 min at 4 °C. The aqueous phase containing the RNA was transferred into a collection and mixed with 75% ethanol (1:1, v:v). mRNA was then purified using PureLink® RNA Mini Kit (LifeTechologies™) following the manufacturer’s instructions. RNA eluates were stored at −80 °C until use. The concentration of each RNA sample was determined by NanoDrop® spectrophotometer ND-1000 (NanoDrop Technologies, Wilmington, DE). The quality of RNA samples was assessed with the Agilent 2100 Bioanalyzer (Agilent Technologies Inc., Santa Clara, CA).

### Immunoprecipitation of muscle exosome subpopulation

Polymer-precipitated exosomes were immunoprecipitated using anti-CD63 magnetic beads (Invitrogen™) overnight according to the manufacturer’s instructions. Magnetically captured beads were then washed 3 times in PBS and CD63 positive exosomes were eluted in 4× NuPAGE™ LDS sample buffer. Samples were then used for western blot analyses as described above.

### Exosome functionality assessment

The exosomes were labeled with the PKH26 kit (Sigma-Aldrich®). Briefly, 100 μl of Diluent C was added to the exosome suspension and labeled with 100 μl of 4 μM PKH26 solution. After 5 min of incubation, samples were washed 3 times in PBS using a 100 kDa Amicon® filter column and centrifuged at 12,000×*g* at 4 °C for 15 min. Muscle exosomes extracted from 3000 differentiated myoblasts were either added to 3000 human iPSC-derived motor neurons or to 3000 differentiated human myoblasts. Human iPSC-derived motor neurons were differentiated from human neuron progenitors as described in [40]. Uptake of muscle exosomes by recipient cells was observed after 24-h incubation in living cells using an Olympus IX170 inverted microscope, with a 40×/0.60 Ph2 objective equipped with an AxiocamMR camera.

### Statistical analysis

All values are presented as means ± SD. ANOVA 1 Factor followed by Tukey post-hoc test was used to compare the differences between the different cell densities conditions. Differences were considered to be statistically different at *P* < 0.05.

## Results and discussion

### Determination of the window of cell divisions suitable to study the muscle secretome in non-senescent stages

**Fig. 1:**
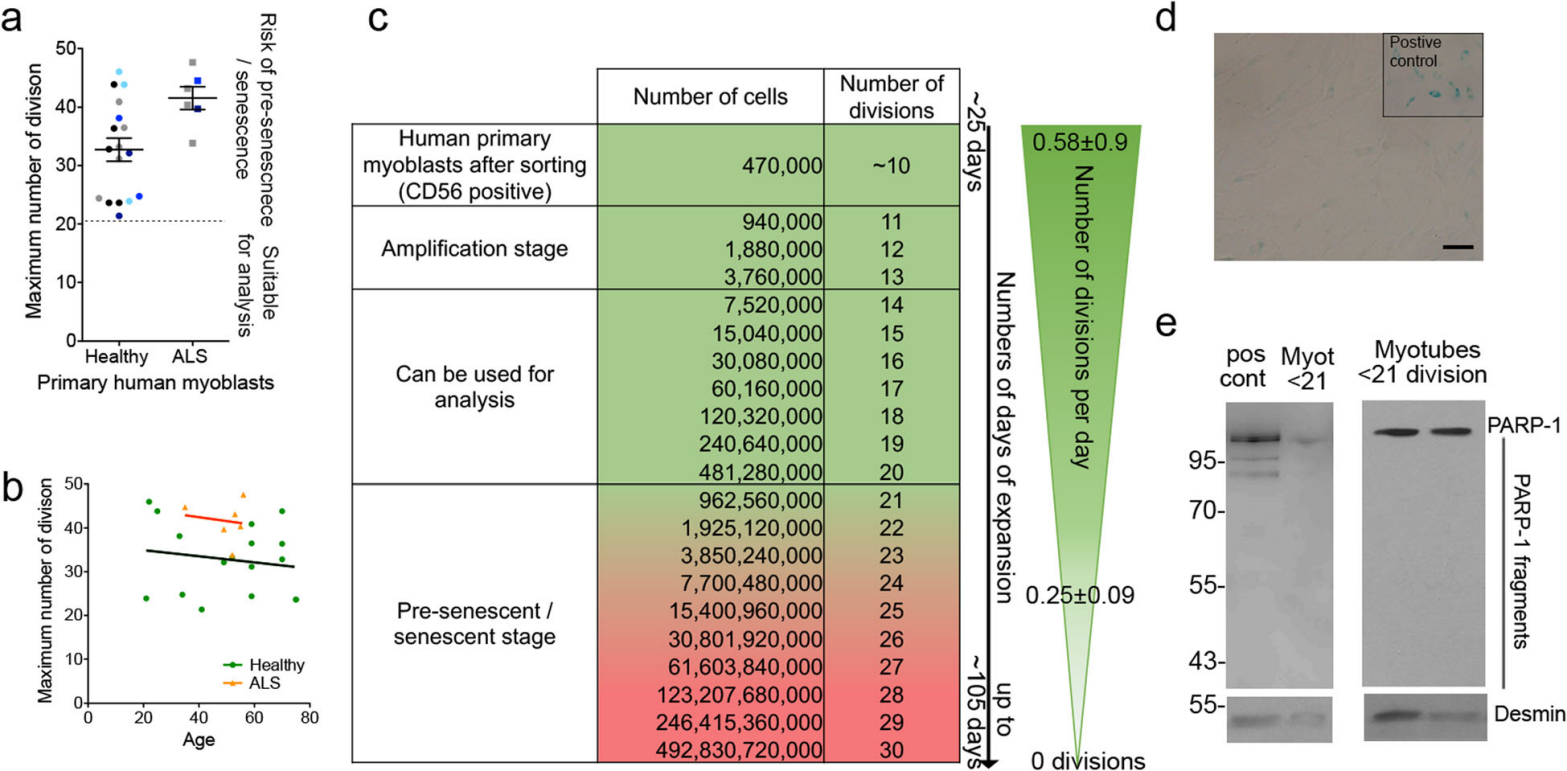
Maximum number of divisions reached by primary human muscle cells, and the number of divisions required to obtain sufficient cell numbers: **a** Distribution of the maximum number of divisions that human muscle cells can execute. Each point represents one sample. Based on this number, a safe window to analyze fully active and proliferative muscle cells is under 21 divisions. Light blue: age 20–30, dark blue: age 30–40, gray: 40–50, black: 50–75 years old. **b** Absence of correlation between age and the maximum number of divisions. **c** Table showing the number of primary muscle cells obtained at different phases of cell culture. Typically, 470,000 CD56+ve muscle cells can be purified from a muscle biopsy culture after ~10 divisions (first row, light green). The number of cells after each division and the number of divisions is given by row. Pink indicates the pre-senescence stage (based on the data in **a**) when cells may start to slow down their capacity to proliferate and then senesce (and rate of division drops from an average of 0.58 to 0.25). Importantly, for some subjects, the cells will not reach 30 divisions, as shown in plot 1a. Typical measurements of the number of days of expansion and of the average number of divisions per day are given on the right side of the table. **d** Myoblasts under 21 divisions were negative for beta-galactosidase. Top right panel: positive control of senescent cells positive for beta-galactosidase. Scale bar = 100 μm. **e** No cleaved PARP-1 was observed by Western blot, suggesting that myoblasts under 21 divisions do not show any sign of necrosis nor apoptosis

Previously published studies on muscle cells using the ultracentrifugation method [5, 32] showed that 250 × 10^6^ cells were needed in order to have enough material for 1 single data point for proteomic and transcriptomic analysis. However, primary muscle cells can only execute a limited number of divisions, ~30–40 divisions with several outliers as low as 22 divisions (Fig. 1a, b, [31]), before they stop dividing and become senescent. The maximum number of divisions is not age-dependent, which is consistent with our previous study showing that the myoblasts extracted from subjects have the same proliferative capacity as myoblasts extracted from young adults [41] (Fig. 1b). Senescent cells can secrete factors including exosomes that can impact surrounding cells as observed with senescent endothelial cells [42], cerebroendothelial cells [43], or fibroblasts [44]. In order to avoid potential artifacts arising from cells that are nearing, or have reached senescence, we suggest that myoblasts under 21 divisions should be used to study the muscle secretome (Fig. 1), and we, therefore, sampled cells within this window for all subsequent experiments.

### Optimization of the muscle cell culture conditions

**Fig. 2:**
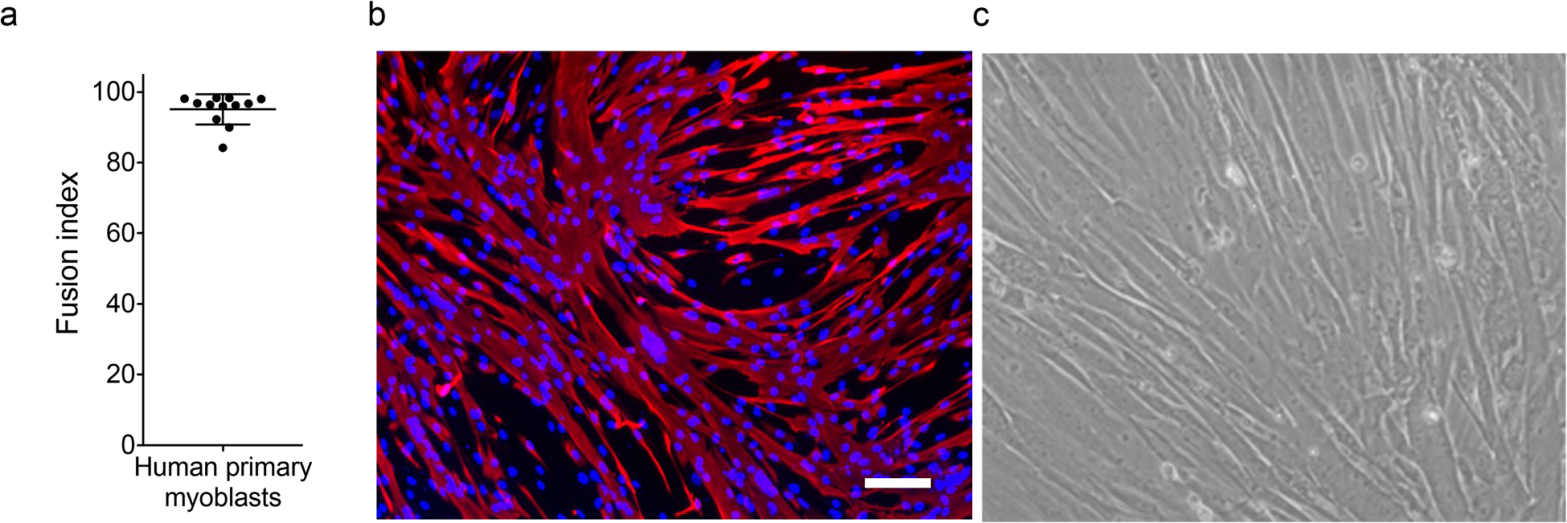
Myoblasts at under 20 divisions differentiate efficiently and are not senescent**.** A total of 12 separate primary cell lines were cultured to under 21 divisions. **a** Dot-plot showing the percentage of primary human myoblasts fused into myotubes for 12 separate cell cultures, with an average fusion index calculated as 95.14% ± 4.28. **b** Representative images of myotubes positive for myosin heavy chain (in red), a marker of differentiation. Scale bar = 100 μm. **c** Over 80% of the flask is covered and no obvious signs of cell death are observed. Scale bar = 100 μm.

Muscle exosomes were extracted from myoblasts that had undergone between 16 and 20 divisions, seeded at a density of 33,400 cells.cm^−2^, and that were differentiated into myotubes for 3 days. Ninety-five percent of the myoblasts were differentiated into myotubes in DMEM after 3 days (Fig. 2a–c), covering over ~80% of the petri dish (Fig. 2 d,e). Differentiated myoblasts were negative for Beta-galactosidase (Fig. 2d), confirming that they were not in a senescent state. Neither necrosis nor apoptosis was observed as PARP-1B was not cleaved (Fig. 2e). These data suggest that human muscle myoblasts which have made less than 20 divisions can differentiate efficiently into myotubes, are not senescent, and are therefore suitable for the study of the myotube secretome.

### Optimization of muscle exosome extraction

**Fig. 3:**
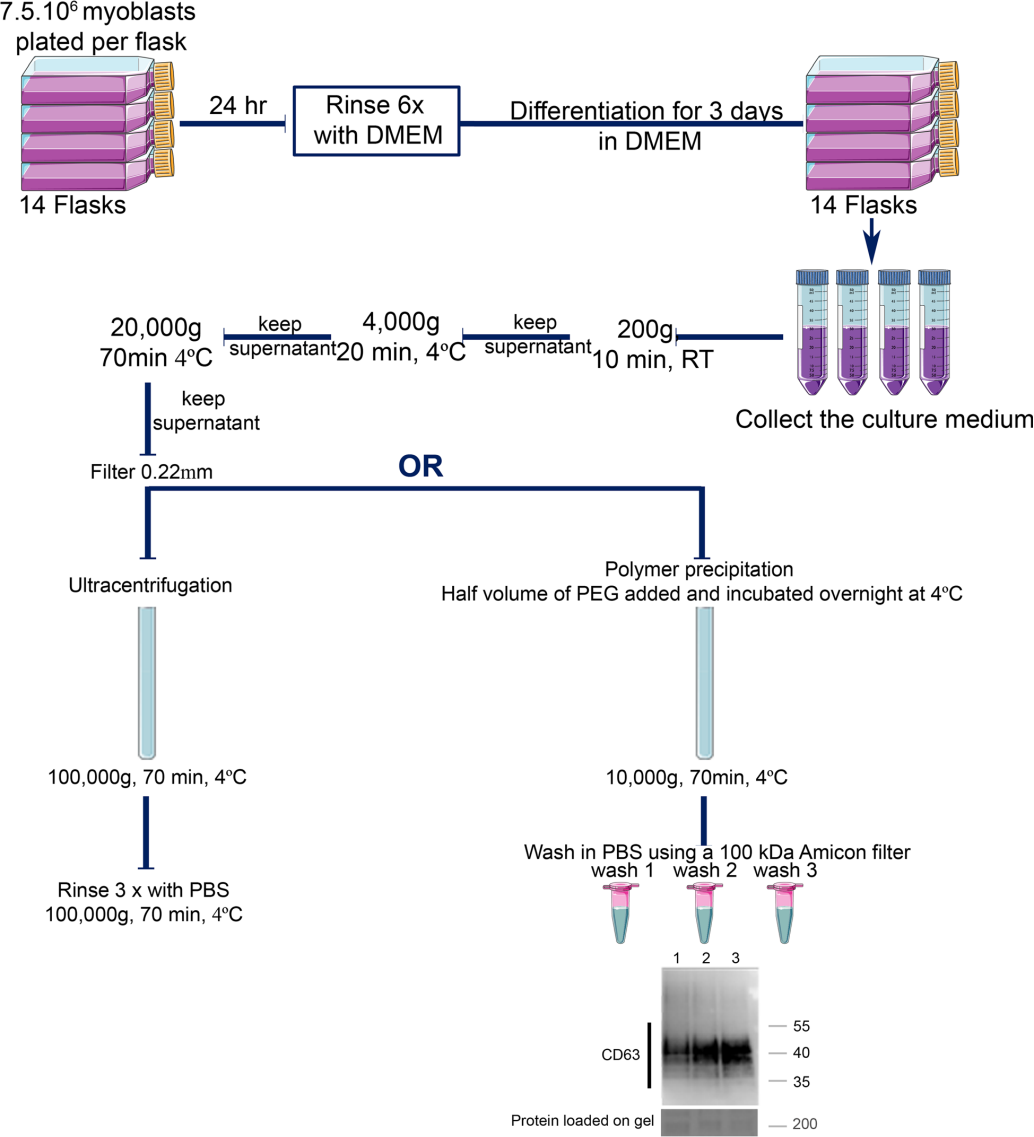
Schema summarizing the protocols used to extract muscle exosomes from the primary human myotube culture medium, using either the ultracentrifugation or the modified polymer-precipitation strategy. For a single data point, 14 flasks of 225 cm^2^ are plated with 7.5 × 10^6^ myoblasts. After 24 h, once the myoblasts have attached to the flask, they are rinsed 6 times in DMEM and then differentiated into myotubes by cultivating them in DMEM. After 72 hr, the conditioned medium is collected for muscle exosome extraction. After removing dead cells (200 g, 10 min, RT), cell debris (4000 g, 20 min, 4 °C), and ectosomes (20,000 g, 70 min at 4 °C, and filtered at 0.22 μm), the cleared media is subjected to exosome extraction either by the ultracentrifugation protocol or by a modified polymer-precipitation protocol. Ultracentrifugation is at 100,000*g* (70 min, 4°C), which is followed by washing the pellets three times with PBS (100,000 g, 70 min, 4°C). The subsequent pellet is then either resuspended in 100 μl of PBS or in NuPAGE™ LDS sample buffer for western blot. For the modified polymer-precipitation protocol, the polymer is added at half the volume of the pre-cleared media and incubated overnight at 4 °C. The mix is then centrifuged at 10,000*g* for 70 min at 4 °C. The subsequent pellet is then washed 3 times in PBS using a 100 kDa Amicon® filter column. Western blot shows the rescue of the epitope CD63 after 3 washes in PBS

Myoblasts were seeded at 7.5 × 10^6^ cells per 225 cm^2^ flask. Due to the large volume of medium (250 ml per sample) required for ultracentrifugation, a total of 14 flasks, thus 100 million differentiated myoblasts, were cultured per data-point and per experiment to compare the efficacy of the ultracentrifugation and polymer-based precipitation protocols. Myotubes were maintained in conditioned media for 3 days. After pre-clearing the media, as described in the materials and methods and as shown in Fig. 3, exosomes were extracted using either the ultracentrifugation strategy or polymer-based precipitation. Previous publications showed lower exosomal protein detection (e.g., CD63) by Western blot using the polymer-based precipitation compared to ultracentrifugation, despite observing a greater number of vesicles by NanoSight using polymer-based precipitation [28, 45]. Based on these publications, we suspected that the polymer matrix was hiding epitopes. After rinsing the exosome extracts 3 times with PBS in 100 kDa Amicon® filter columns, the accessibility of antibodies to epitopes was rescued (Fig. 3).

### The ultracentrifugation-based and modified polymer-based precipitation approaches both extract exosomes from conditioned cultured media of primary human myotubes, but the polymer-based approach is more efficient

**Fig. 4:**
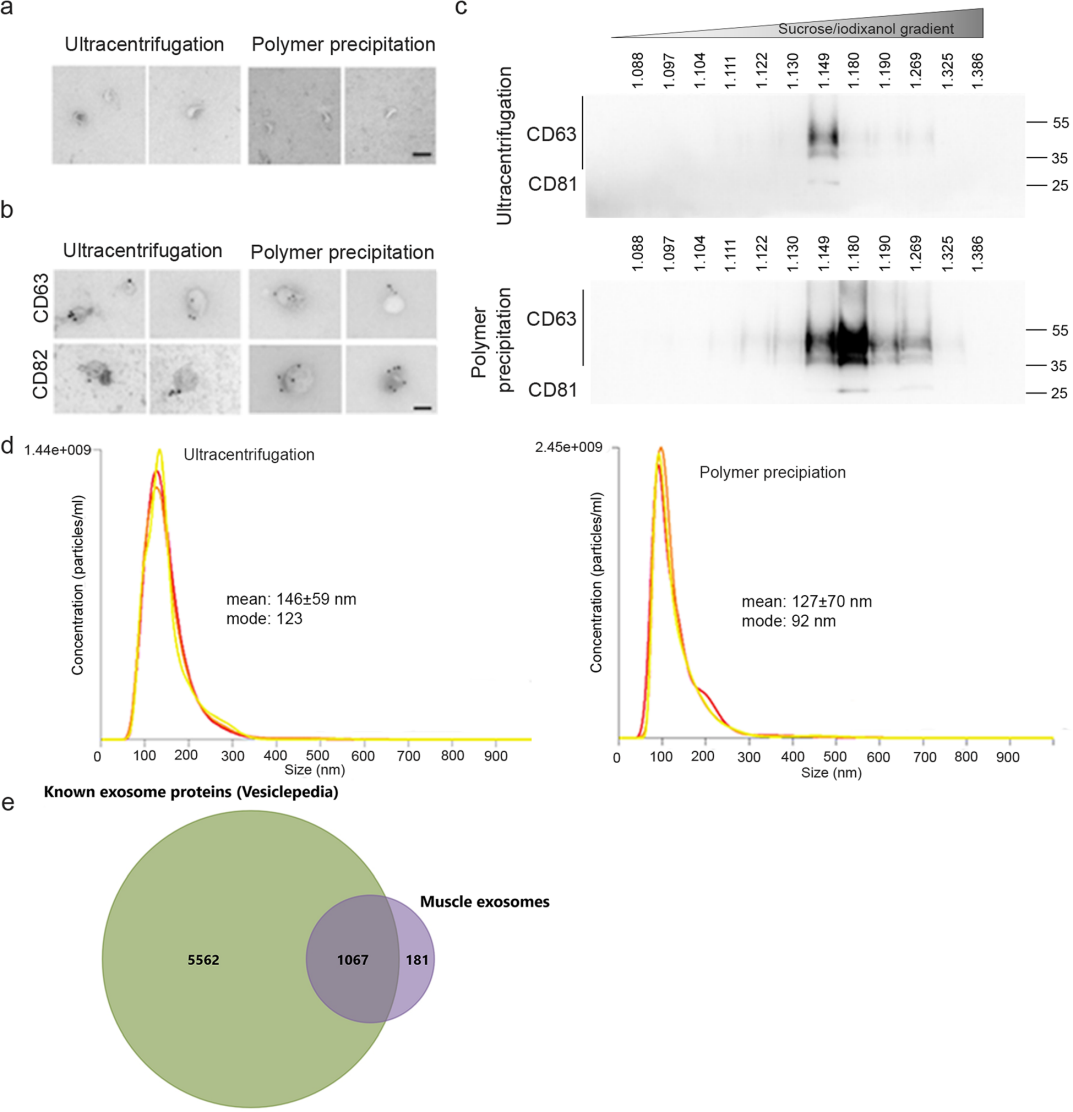
Validation of exosome extraction strategy**.** For each experiment, exosomes were extracted from the culture medium of 100 × 10^6^ myoblasts differentiated into myotubes for 3 days using either the ultracentrifugation or the polymer precipitation. The culture medium was non-supplemented DMEM (without serum). **a** Cup-shaped vesicles were observed by electron microscopy with both extraction protocols. bar = 100 nm. **b** Both extractions show vesicles that are positive for CD63 and CD82 by electron microscopy. Bar = 100 nm. **c** Exosome extracts were loaded on iodixanol gradients as described in material and methods. Western blot results are shown for CD63 and CD81 in twelve fractions for the iodixanol gradient. Top panel: exosomes extracted by ultracentrifugation. Bottom panel: exosomes extracted by polymer-based precipitation. Exosomes were detected at a density of 1.15–1.27 g.ml^−1^. **d** Nanosight analyses show similar-sized vesicles using both strategies, from 100–200 nm, with a greater number of particles being detected when using the polymer extraction. **e** Proteomic analysis of muscle exosomes. Venn diagram showing the overlap between muscle exosomes and proteins known to be detected by mass spectrometry in exosomes (Vesiclepedia, Exocarta database [36–39])

Exosomes secreted by 100 × 10^6^ differentiated myoblasts and extracted using either ultracentrifugation or polymer-based precipitation show the same cup-shape structure by electron microscopy (Fig. 4a) and are positive for CD63, CD82 (Fig. 4 b,c) ,and CD81 (Fig.4c), and float at the same density (Fig. 4c). Similar sized vesicles were observed by electron microscopy and by NanoSight analysis (Fig. 4d). Importantly, the ultracentrifugation strategy was far less efficient than the polymer precipitation to extract exosomes as shown in Fig. 4c and d. A proteomic analysis revealed that the protein profile of the muscle vesicles extracted using the modified polymer-based precipitation is enriched in proteins known to be present in exosomes (Fig.4d) as given by Exocarta [36–39].

### Working with >30 times fewer myoblasts, the modified polymer precipitation strategy still efficiently extracts vesicles that can be used for follow up experiments

**Fig. 5:**
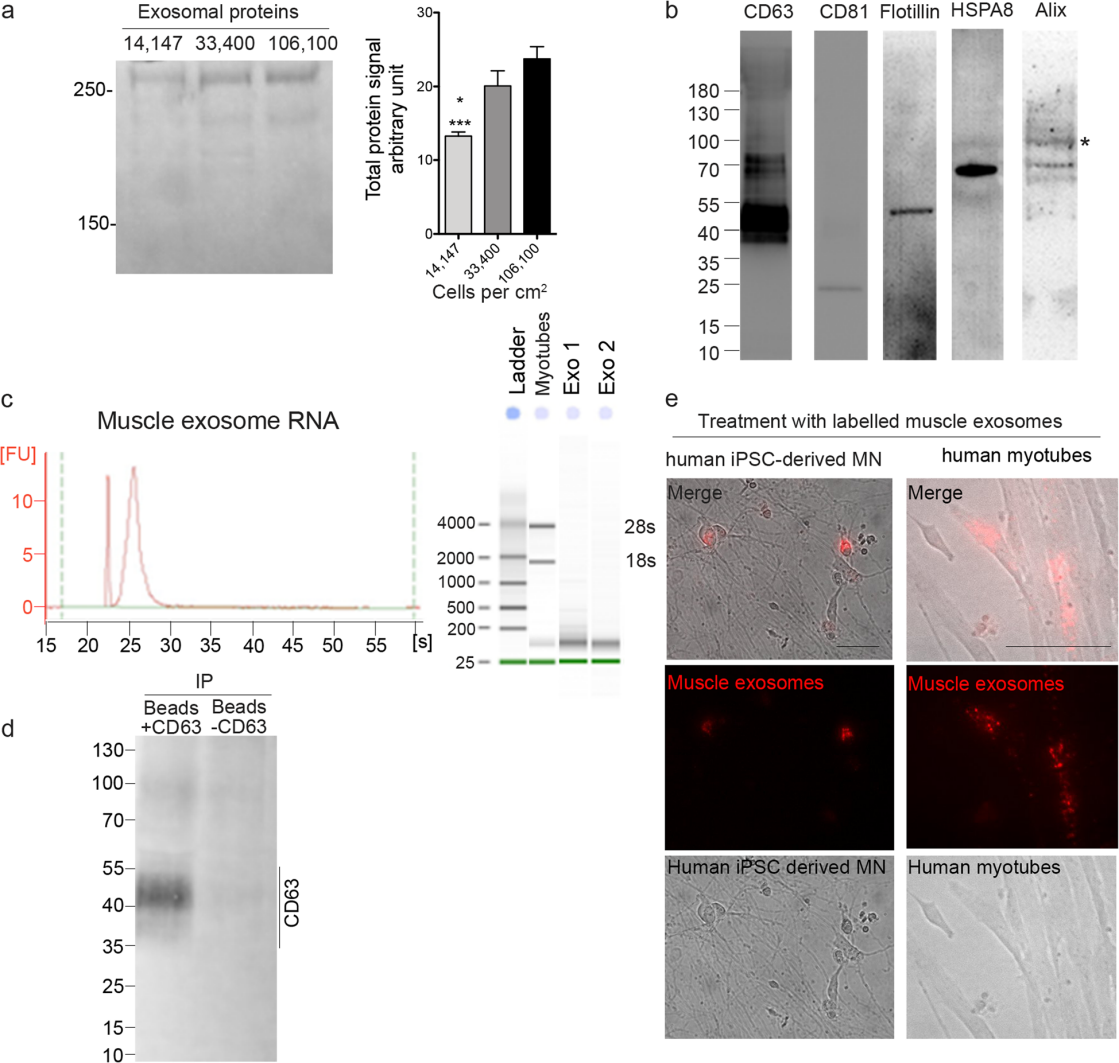
Polymer-based precipitation efficiently extracts functional exosomes from 7.5 × 106 cells. **a** SDS-page protein quantification showing that the greatest efficiency in terms of exosomal protein per cell plated was obtained when exosomes were extracted from differentiated myoblasts at a density of 33,400 cells.cm^−2^. Differentiated myoblasts were plated at 14,147 (lane 1), 33,400 (lane 2), or 106,100 (lane 3) cells per cm^2^. Right panel: representative SDS-page stained with Coomassie. Left panel: protein concentration measurements in secreted exosomes from cells plated at a different density. *, ***, *P* < 0.05 and *P* < 0.001, significantly different from 33,400 and 106,100 cells.cm^2^. (*n* = 4, 3, 4 per condition). **b** Muscle exosomes were positive for CD63, CD81, Flotillin, HSPA8, and Alix. **c** mRNA was detectable with a clean profile from polymer-precipitated exosomes of 7.5 × 10^6^ differentiated myoblasts. No 18 s and 28 s RNA were detected, indicating that there were no RNA contaminants from dead cells. Inset panel: Representative electrophoresis obtain with Agilent 2100 Bioanalyzer for myotubes and exosome RNA extract. **d** Western blot showing that polymer precipitated exosomes from 7.5 × 10^6^ differentiated myoblasts can be used to pull down a specific subpopulation such as CD63 positive exosomes (+/-CD63 = with/without anti-CD63 antibody). **e** Polymer-precipitated exosomes (pre-stained with PKH26 following extraction; red channel) were capable of integrating into myotubes or into iPSC motor neuron cells

Previous publications suggested that cell density may affect exosome secretion [46]. We thus tested different densities of differentiated myoblasts per cm^2^ and observed that the optimal conditions were 33,400 cells.cm^−2^ (Fig. 5a), thus 7.5 × 10^6^ myoblasts for a 225 cm^2^ flask. Exosomes secreted by muscle cells were positive for CD63, CD81, Flotillin, HSPA8, and Alix (Fig. 5b). Exosomes extracted from 7.5 × 10^6^ differentiated myoblasts could be used to explore exosome mRNA content (Fig. 5c) and could be used to isolate a specific subpopulation of exosomes such as CD63-positive vesicles (Fig. 5d). In addition, polymer-precipitated exosomes can be stained with PKH26 and applied to recipient cells such as myotubes or iPSC-derived motor neurons (Fig. 5e).

## Conclusion

Although emphasis has been given to the role of the muscle tissue environment in regeneration (e.g., in parabiosis experiments [47, 48]), very little is known about the secretome of human muscle cells. The role of muscle as a secretory endocrine organ has been recently proposed and a number of studies have characterized the secretory profiles of muscle cells [5, 7, 32, 49, 50], but the role of muscle vesicles is an underexplored field, as is the putative cross-talk between different cell types. Exploring the content and function of vesicles secreted by purified human myoblasts will improve our understanding of how muscle communicates with its environment in different physiological (e.g., aging) and pathological contexts (e.g., neuromuscular disorders, cachexia associated with cancer, etc.) [51–54]. It may also provide new insights regarding the pathological mechanisms underlying such conditions and may help in the identification of novel biomarkers and novel therapeutic targets for diseases.

Only a small number of human muscle cells can be obtained from muscle biopsies and these cells have a very limited capacity to divide. These caveats, along with the fact that muscle cells do not secrete large quantities of vesicles—consistent with muscle accounting for up to 50% of body mass—reinforce the importance of identifying strategies that allow for the most efficient extraction of muscle vesicles from a small quantity of starting material.

Large amounts of starting material are required when using the ultracentrifugation-based technique [55], especially when there is an intention to perform downstream OMICS studies (e.g*.*, proteomic, transcriptomic, metabolomic analyses; 250 × 10^6^ muscle cells for one replicate [5]). Several commercial kits have been developed to improve isolation efficacy and speed. The purity of vesicles isolated using these kits is often questioned in comparison to the ultracentrifugation methodology, especially when extracting from serum/plasma [56, 57], but also in the in vitro context [58, 59]. However, it is important to note that while these studies do adhere strictly to the manufacturers’ instructions for the usage of the kits, they often fail to carry out identical sample preparations prior to the comparison—for example, carrying out centrifugations and/or filtration steps to remove microvesicles and other contaminants before ultracentrifugation but neglecting to do so before using the kits. This, together with the epitope hiding property of the polymer that is discussed below in the context of additional rinsing steps, may largely account for differences in observed contamination rates.

In the present study, muscle exosomes are extracted from differentiated human myoblasts that have been cultured in non-supplemented DMEM. This ensures that exosome preparations isolated using this method are fully depleted of any potential contaminants from culture medium additives such as fetal bovine serum. Furthermore, differentiated myoblasts cultured under these conditions undergo neither necroptosis nor apoptosis (current paper, [60]). When collecting the conditioned media, differential centrifugation steps and a filtration step are included to remove potential cell debris, apoptotic vesicles, and microvesicles. All of these precautions are carried out prior to the addition of the polymer solution, thus eliminating most, if not all potential contaminants and ensuring a highly purified isolation process, and we recommend that such steps are included no matter which subsequent exosome isolation method is used.

The absence of medium supplementation and the lack of necroptosis and apoptosis mean that the culture medium of differentiated human muscle cells is a non-complex sample, and is therefore well-suited to the protocol described here, as opposed to the serum which includes many different types of the vesicle and a relatively complex molecular milieu, thereby making it difficult to isolate exosomes by size and density alone, and requiring additional approaches such as exosome pull-down to maximize purity [61, 62], but leading to the analysis of a specific circulating exosome subpopulation.

Looking at the literature, we noticed that the polymer kit consistently led to a greater number of vesicles detected by NanoSight [56, 59, 63], and yet led to a reduced detection of exosomal markers by Western blot [28, 45, 56, 59, 63, 64]. Interestingly, Rider et al. while optimizing a polymer to extract extracellular vesicles showed that rinsing of exosomes that had been precipitated using the polymer resulted in an increase in exosome markers detected by Western blot [28]. Based on that study, we decided to use 100 kDa Amicon® filter columns to add extra washes after precipitating the vesicles from pre-cleared media. These additional steps removed the surplus of the polymer [65], thereby rescuing the detection of exosomal markers (Fig. 3), and likely have the additional advantage of removing any cytokines [58] secreted by muscle cells. These extra rinsing step may also improve the functionality of the exosome-like vesicles, for experiments involving the incorporation of vesicles into recipient cells (Fig. 5e).

Pre-clearing the culture medium followed by polymer precipitation and three PBS washes allows the extraction of exosome-like vesicles while using 33 times less starting material than what is needed when the ultracentrifugation protocol is used, and the quality and functionality of extracted exosomes is retained. The option of being able to carry out proteomic and functional analyses on exosomes while requiring much fewer cell numbers as a starting point is a critically important asset especially when dealing with primary cell cultures that quickly senesce [66, 67].

## Data Availability

All data and materials will be available on demand.
